# Educational standards for training paramedics in ultrasound: a scoping review

**DOI:** 10.1186/s12873-017-0131-8

**Published:** 2017-06-17

**Authors:** Ben Meadley, Alexander Olaussen, Ashleigh Delorenzo, Nick Roder, Caroline Martin, Toby St. Clair, Andrew Burns, Emma Stam, Brett Williams

**Affiliations:** Department of Community Emergency Health and Paramedic Practice, Monash University – Peninsula Campus, PO Box 527, McMahons Road, Frankston, VIC 3199 Australia

**Keywords:** Out-of-hospital, Paramedic, Ultrasound, Education, Training

## Abstract

**Background:**

Paramedic-performed out-of-hospital ultrasound is a novel skill that has gained popularity in some services in recent years. In this setting point-of care ultrasound (POCUS) can provide additional information that can assist with management and guide transport to the most appropriate facility. We sought to explore the different educational approaches used for training paramedics in ultrasound in the out-of-hospital setting.

**Methods:**

Ovid MEDLINE, EMBASE, EBM Reviews, The Cochrane Library, CINAHL plus, The Monash University Research Repository and the British Thesis Library were searched from the 1^st^ of January 1990 to the 6^th^ of April 2016. Google Scholar was searched and reference lists of relevant papers were examined to identify additional studies. Articles were included if they reported on out-of-hospital and POCUS educational approaches for paramedics.

**Results:**

A total of 2002 unique articles were identified of which 18 articles met the inclusion criteria. Most articles reported combined cohorts of emergency providers with varying years of experience though most operators were POCUS naïve. The most common clinical assessment for which paramedic POCUS curricula was described was the focused assessment sonography for trauma (FAST) examination. Education programs varied from two-minutes to two-days with all studies including both didactic and practical training.

**Conclusion:**

Education programs for POCUS for paramedics vary considerably, and do not appear to align with qualification level or clinical experience. Further research investigating education and subsequent clinical application of POCUS by paramedics is required, as well as prospective, outcome based studies in order to measure the clinical utility of out-of-hospital POCUS.

## Background

The modern-day Emergency Medical Services (EMS) system is a complex network of coordinated services. Due to the challenging environment and ever-changing nature of work encountered by paramedics, the profession is continually expanding to include a greater range of clinical skills [1]. For example, recent advances have included the addition of point-of-care ultrasound (POCUS) to assist with improved patient outcomes, clinical decision making and triage [2–4]. Balancing over-triage and under-triage is important for the health-care system as a whole and the patient as an individual [5]. Clinical diagnoses alone are often insufficiently sensitive, leading to misdiagnoses and under-triage of some clinical conditions. In the out-of-hospital setting POCUS can provide additional information, such as earlier detection of intra-abdominal free fluid in patients with blunt trauma, and pericardial effusion in patients with penetrating thoracic trauma [2, 3]. This information can assist with management strategies, guide transport to the most appropriate facility, and potentially expedite time to definitive intervention [3].

Clinical applications of POCUS in the out-of-hospital setting include enhancing assessment of a wide range of patient cohorts, including but not limited to medical and traumatic patients [2]. For example, the Focused Assessment Sonography for Trauma (FAST) exam is used to detect free fluid in the intra-abdominal compartment [4]. The application of FAST by paramedics, emergency physicians and flight nurses has been reported with greater sensitivity and specificity, compared to physical assessment alone, when assessing for hemoperitoneum in patients with penetrating and blunt trauma [6, 7].

Moreover, the extended FAST examination (eFAST) also includes thoracic assessment to detect for haemothorax and pneumothorax [8]. This is a time effective procedure particularly when faced with the challenges of auscultating breath sounds in the out-of-hospital environment, such as in a moving ambulance or during aeromedical transport. A diagnostic accuracy of over 90% when using POCUS to assess for pneumothorax has been reported, suggesting POCUS may be superior to other clinical assessment tools [8]. Correct identification of the presence or absence of pneumothoraces can help guide urgent interventions during resuscitation. Alternatively, this may also prevent unnecessary procedures such as pleural decompression thereby avoiding an invasive procedure that carries potential complications [9, 10]. Other applications of POCUS performed by paramedics include modified cardiac echocardiography to assess for pericardial fluid and tamponade physiology in the setting of trauma and assessing intra-arrest cardiac wall motion [2, 3].

The ability to accurately perform and interpret sonography is likely dependent on appropriate training and education of paramedics [2]. General paramedic training can vary considerably, from vocational based-training through to formal tertiary education at the postgraduate level. With regard to demonstrating competence in POCUS, dependence on background education appears most apparent in EMS systems that use non-physician providers, including paramedics and nurses [2]. Most studies report on EMS providers from a range of clinical backgrounds, and evidence pertaining to the clinical utility of paramedic use of POCUS is limited. A systematic review identified multiple training programs with various delivery methods, duration, and assessment [9]. Similarly, El Sayed et al. [2] and Nelson et al. [4] also discussed POCUS curricula for EMS providers in the out-of-hospital setting. These reviews report POCUS to be feasible and time effective with successful application. However, variations in training duration, and the quality of POCUS examinations by different providers are yet to be compared [4]. Presently, it appears there is no agreed curricula standards for the training of paramedics in the provision of out-of-hospital POCUS, making it difficult to draw conclusions regarding the optimal training approach for paramedics.

With the increasing use of out-of-hospital POCUS to assess a number of clinical conditions, it is important to gauge the different methods by which POCUS is currently being taught to paramedics. This will guide which methods suit various EMS agencies with regards to affordability, applicability, and effectiveness. This scoping review aims to explore the different educational approaches used for training paramedics in POCUS in the out-of-hospital setting.

## Methods

We desired to map the available literature relating to paramedics and POCUS education. We therefore chose a scoping review methodology in order to provide a broader understanding of this topic, which can then be narrowed down to a specific research question and systematic review [11, 12]. In accordance with scoping review practice we included both peer- and non-peer reviewed research, as well as grey literature [12]. We followed the six stage methodology developed by Levac et al. (2010) [12].

1. *Identify the Research Question*
  The research question guiding this scoping review was: What educational approaches are used for training paramedics in POCUS? This question was decided upon after initial review of the available literature. The question was felt to strike a balance between being broad enough for a wide selection of papers and at the same time focused enough to generate a search strategy [12].
2. *Identify relevant studies*
  The databases Ovid MEDLINE, EMBASE, EBM Reviews, The Cochrane Library, CINAHL plus, The Monash University Research Repository and the British Thesis Library were searched from the 1^st^ of January 1990 to the 6^th^ of April 2016. The Journal of Medical Sonography and the Air Medical Journal were also searched specifically. In addition, a rigorous search of the grey literature was conducted (Google Scholar, Grey Literature Report and GreyNet International) to identify further studies that might have been missed by the electronic search. A combination of Medical Subject Headings (MeSH) terms and keywords relevant to paramedics and out-of-hospital, POCUS and education (Table 1) were combined using Boolean terms as appropriate. Education interventions were defined as anything featuring teaching, training or practical supervision. We define training in this instance any intervention aimed at increasing competency with use of POCUS.

**Table 1:** Summary of search terms combining the 3 search concepts

Paramedics & Out-of-hospital	Ultrasound	Education
emergency medical services/	ultrasound.mp.	Education/
emergency medical technicians/	Ultrasonography/	Teaching/
emergency treatment/	sonography.mp.	education.mp.
emergency medicine/	Ultrasonography, Doppler, Color/	Simulation Training/
ambulances/	Diagnostic Imaging/	training.mp.
air ambulances/	ultraso$.mp.	educat$.mp.
retrieval	sonograph$.mp.	teach$.mp.
HEMS		train$.mp.
first aid/		
military medicine/		
prehospital		
pre-hospital		
paramedic		
ambulance		
out-of-hospital		
out of hospital		
ems		
emt		
emergency services		
emergency medical service		
emergency technician		
emergency practitioner		
emergency dispatch		
emergency despatch		
first responder		
public access defibrillation		
emergency rescue		
emergency resus		
emergency triage		
advanced life support		
community support co-ordinator		
community support coordinator		
emergency care practitioner		
extended care practitioner		
physician assistant
3. *Study selection*
*Inclusion and exclusion criteria*
    Studies were eligible for inclusion if they (i) reported on POCUS educational approaches for paramedics and out-of-hospital, and (ii) were published between January 1990 and the 6^th^ of April 2016. This time period was selected because (i) a preliminary review and intimate knowledge of the field suggested there would not be any relevant articles prior to the year 1990, (ii) cost, and (iii) to only include current and modern methods of education. Studies were excluded if they were not written in English, or if they did not include paramedic providers.
    The data bases were searched by one author (AO). Following the search, duplicates were removed and the titles and abstracts subsequently appraised for eligibility by five independent authors (BM, NR, TSc, CM and BW). The full texts of titles were sourced and reviewed for those studies which were considered potentially relevant. The selection process is described below, see Fig. 1.

**Fig. 1:**
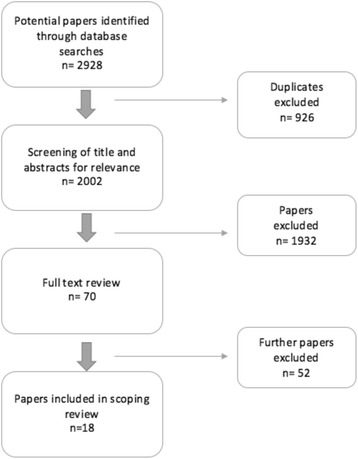
Flow diagram showing the identification of studies assessing POCUS educational approaches for paramedics
4. *Charting the data*
  Charting the data is a ‘narrative way’ or ‘descriptive analytical’ method that is used to extract the data from each study [13]. Table 2 provides an overview of the 18 articles selected for inclusion. The key information from the chosen articles were charted according to a common analytical framework [13]. The main areas of extracted data were participants, educational method (didactic, simulation, clinical), time and resource, and clinical condition researched.

**Table 2:** Characteristics of the studies

Author, year Country	Participants and Number	Operators & experience with US	Aim	Clinical Conditions	Test Population	Methods
Baldaranov et al. 2015 [20] Germany	*n* = 6 Paramedics	US naive	To design and evaluate a dedicated stroke educational program for paramedics including transcranial POCUS.	Stroke	Real patients	Prospective observational study
Booth et al. 2015 UK [14]	*n* = 9 Paramedics	US naive	Assess whether paramedics can be trained to perform & interpret echo	Cardiac arrest	Healthy models	Prospective observational pilot study
Brooke et al. 2010 UK [37]	*n* = 10. Paramedics	Advanced paramedics, US naive	Determine if advanced paramedics can be trained to acquire and interpret quality ultrasound images	Pneumothorax	Pre-recorded video clips	Prospective observational cohort study
Cappa et al. 2015 Arizona [28]	Not reported	ED nurses and paramedics	Determine if a program to train nurses and paramedics to place USGIV’s in the ED decreases the use of central lines.	Peripheral Intravenous access	Not reported	Combined retrospective and prospective observational study
Chin et al. 2013 USA [16]	*n* = 20 Firefighter paramedics	US naive	Determine if EMTs can be trained using Pre-hospital Assessment with US for Emergencies (PAUSE) protocol	Pneumothorax, pericardial effusion & cardiac standstill	Healthy models	Prospective educational intervention study
Heegaard et al. 2004 USA [7]	*n* = 10. Flight nurses & paramedics	>5 years clinical experience	Develop a training program for air medical clinicians using focused POCUS examinations and assess competencies 1 year later.	FAST in medical & trauma cases	Real patients	Prospective observational study
Heiner & McArthur 2010 USA [17]	*n* = 20. EMTs	US naive	Study ability of EMTs to be trained to recognize presence of fractures using portable POCUS	Long bone fractures	Simulation model on turkey leg bone	Prospective observational study
Knapp et al. 2012 USA [22]	*n* = 90 Paramedics 70 EMT-Ps and 20 EMT-Is	Paramedic and Intermediate EMT providers. US naïve.	Determine whether EMS providers at the EMT-intermediate and EMT Paramedic levels can acquire knowledge and skill to operate portable POCUS and achieve high level of accuracy performing cardiac and FAST exams.	Cardiac and FAST	Live standardised patients	Prospective Cohort Educational Study
Lahham et al. 2015 USA [21]	*n* = 4 Paramedics	US naïve	Determine whether paramedics are capable of obtaining cardiac POCUS images and can use these for adequate clinical decision making, as well as identify cardiac activity in cardiac arrest patients.	Cardiac evaluation	Real patients	Prospective educational intervention study
Lema et al. 2014 New York [26]	*n* = 31 paramedics *n* = 2 residents	Naïve US-guided intubation experience	Assess whether paramedics and residents could dynamically identify correct ETT placement in a cadaver model using US.	Correct endotracheal tube placement	Cadaver models	Prospective observational study
Lyon et al. 2012 USA [23]	*n* = 8 (4 critical care flight paramedics & 4 critical care nurses)	Two with previous limited US exposure, none with experience with clinical US	Determine if pre-hospital critical care providers can be trained to determine presence/absence of the sliding lung sign on POCUS.	Pneumothorax	Cadaver models	Blinded RCT
Press et al. 2013 USA [24]	*n* = 33 Helicopter paramedics and flight nurses	Majority had no US experience.	Effectiveness of an EFAST training program	FAST	Both simulated patients (with pathology) and real patients	Prospective observational cohort study
Quick et al. 2016 USA [8]	*n* = 26 flight crew members (flight nurses and paramedics)	In HEMS helicopter with flight crew. US training prior to study commencing.	To evaluate the ability of non-physician aeromedical providers to identify pneumothorax in-flight.	Pneumothorax	Initial training: Healthy models and swine animal models. Study: Real patients	Prospective observational study
Roline et al. 2013 USA [27]	Flight Crew *n* = not reported	In HEMS helicopter with flight crew	Evaluate feasibility of bedside thoracic US in helicopter environment	Pneumothorax	Healthy model in training. Real non-pregnant patients transported by HEMS	Prospective pilot study
Unleur et al. 2011 Turkey [18]	*n* = 4. Paramedics	Senior paramedics working in ED triage. US naive	Accuracy of paramedic performed FAST in ED after trauma	FAST	Real patients	Prospective observational study
Walcher et al. 2010 Germany	*n* = 9. 5 ED doctors & 4 paramedics (403 participants total trained from 2003 to 06)	US naive. US training day then performed on scene.	Evaluate effectiveness of new training course for prehospital FAST (P-FAST)	FAST in trauma patients	Healthy models, models with positive FAST (ascites or peritoneal dialysis), and real patients	Prospective, multi-centre study
West et al. 2014 USA [19]	*n* = 9 paramedics (10 enrolled but one pulled out on examination day)	Paramedics with field experience but US naïve	Evaluate the accuracy andtime taken to perform multiple FAST exams in a simulated MCI setting.	FAST in trauma patients during MCI	Healthy models and models with positive FAST (peritoneal dialysis patients)	Single-blinded RCT
Vitto et al. 2015 USA [25]	*n* = 15 flight nurses and paramedics	. US naïve	Evaluate the ability of flight nurses and paramedics to learn and retain U/S for use during flight and ground transport.	Not Reported	Healthy models and US simulations using US simulator	Prospective observational cohort study
5. *Collating, summarising and reporting the results*
  A total of 18 articles were included, comprising 11 prospective observational studies, 2 prospective educational intervention study, 1 single-blinded randomised controlled trial, 1 prospective educational cohort study, 1 prospective multi-center study, 1 blinded randomised controlled trial, and 1 combined retrospective and prospective observational study. Results are presented below and a summary is tabulated in Tables 2 and 3.

**Table 3:** Educational focus of the studies

Study	Education Method	Educational Focus	Duration	Education details	Results
Baldaranov et al. \2015 [20]	Didactic (online) + practical	Application & interpretation	2-months	A course of 2-months. Web based curricula designed in two parts: (1) theoretical and (2) real life training under neurological supervision.	Study is ongoing.
Booth et al. 2015 [14]	Didactic & practical	Application, knowledge & interpretation	2 h of lectures & 4 h of practical and simulation	Paramedics were trained to under-take two attempts at a subxiphoid and parasternal long axis view and to assess images for the following: movement, function, rhythm, fluid and chambers. This systematic approach focused on evaluating the presence and quality of cardiac movement, and to detect conditions amenable to therapeutic intervention. To simulate actual OHCA, some scanning was performed on the floor. Participants completed a pre-course and post-course questionnaire.	88% obtained successful views during timed 10s pulse check Theoretical knowledge improved (54% pre-course to 89% post course)
Brooke et al. 2011	Didactic & practical	Application, knowledge & interpretation	2-day education & training program	Participants reviewed the pathophysiology and management of patients with pneumothorax before learning to differentiate the normal and abnormal sonographic appearance of the lungs. Lung US was taught in a systematic manner. Following the program, the participants were assessed by (1) the ability to detect the presence or absence of a pneumothorax using 30 prerecorded lung US video clip images and (2) an OSCE.	All paramedics passed examination with standard judged to be equivalent to that expected of candidates in thoracic module of College of Emergency Medicine level 2 US program.
Cappa et al. 2015 [28]	Not Reported	Not Reported	Not Reported	Not Reported	A non-statistically significant decrease (9.2%) in the use of central lines in the ED following implementation of paramedic and nursing-led ultrasound program.
Chin et al. 2013 [16]	Didactic & practical	Application & interpretation	1 h lecture & 1 h practical	Paramedics received training on the basics of ultrasonography, the PAUSE protocol, image acquisition, and basic image interpretation, followed by a 1-h hands-on session. An Emergency Physician trained in bedside US demonstrated the following views on a human model: a thoracic view of the pleural interface of the lung, a subxiphoid cardiac view, and a parasternal long cardiac view. Participants were then assessed by (1) image recognition and (2) the ability to acquire an adequate view of the left and right pleural interfaces and one view of the heart without assistance.	Average score 9.1/10 on image recognition test 6 paramedics were unable to identify cardiac standstill. 100% of images acquired by paramedics were satisfactory to evaluate PTX 55% of paramedics obtained satisfactory cardiac views
Heegaard et al. 2004 [7]	Didactic & practical	Application & interpretation	7 h training program followed by 8 h of hands-on supervised training in ED	Introduction to US, physics and the use of US within air medical practice. This was followed by information on echocardiography, abdominal US, pelvic and obstetrical US, and the FAST examination with a demonstration. The 3-h practical session involved individual instruction before each clinician performed a further 8-h in an ED on emergency patients. In addition, learning resources were made available via a website. At the end of 6 weeks a final training session was provided and skills were assessed. This included (1) a written test and (2) a practical imaging test. These same tests were administered 1 year from the initiation of the program.	US for pericardial effusion: sensitivity & specificity 100% (1/86 cases positive), no false negative or false positive cardiac US Abdominal trauma cases: sensitivity 60%, and specificity 93%. Immediately post-course questionnaire score of 82% vs. percentage when administered 1 year later - 71%
Heiner & McArthur 2010 [17]	Didactic & practical	Application & interpretation	2- min orientation/training	Participants received a 2-min standardized orientation and training session ensuring familiarization with examination of the semi-opaque fracture model. They then sonographically evaluated the 5 completely opaque models.	Sensitivity of 97.5% and specificity of 95.0% across 5 different fracture patterns.
Knapp et al. 2012 [22]	Didactic & practical	Application & interpretation	1 h online home based study program followed by 4 h training program	A four- hour training program that consisted of a didactic lecture, practice scanning, and testing scenarios. All participants underwent a pre- and post-training written test. The final testing scenarios included one normal and abnormal cardiac, and one normal and abnormal FAST. All scenarios were performed on live standardized patients and graded in an OSCE format.	Average score on the pre and post-test was 73% and 95% respectively (*p* < 0.0001). EMS providers (*n* = 90) scored on average 98.9 points out of 100 on the OSCE testing stations. EMT-Ps (*n* = 70) scored, on average 98.9 points out of 100 on the OSCE stations. Average score for EMT-Is (*n* = 20) was 99.1 points out of 100 on the OSCE stations.
Lahham et al. 2015 [21]	Didactic & practical	Application & interpretation	3-h session on POCUS that included didactics, hands-on training and a final test.	A three-hour session on POCUS including didactics, hands-on training and a final test was conducted. Participants then used POCUS in a clinical setting during dispatch calls and saved scans related to: chest pain, dyspnea, loss of consciousness, trauma, or cardiac arrest. The scans were later evaluated by two independent ultrasound fellowship-trained emergency physicians.	Paramedics were able to obtain adequate scans 89% of the time. Two scans were considered of inadequate diagnostic quality. Two cardiac arrest studies were reported and paramedics correctly identified both of these cases as cardiac standstill.
Lema et al. 2014 [26]	Didactic & practical	Application & interpretation	10 min lecture and hands-on session	Subjects intubated four cadavers without POCUS guidance and were assessed for correct tube placement by an emergency physician. All participants then underwent a 10-min lecture and hands-on session about POCUS identification of tracheal versus esophageal tube placement. Participants then intubated four cadavers using POCUS guidance and were assessed for correct tube placement.	Correct tube placement improved from 87.1% (*n* = 132) without POCUS to 95.3% (*n* = 128) with POCUS guidance (*p* = 0.018).
Lyon et al. 2012 [23]	Didactic & practical	Application & interpretation	25 min instructional session	A cadaver was used as a model for demonstrating the presence or absence of the SLS. A total of 6 intubations, yielding a total of 48 trials, were performed. With bag valve ventilation and endotracheal intubation, the pleural movements of the cadaver result in the appearance of the SLS. When intubated in the esophagus, bag valve ventilation results in no pleural movement and, thus, no SLS. The cadavers were randomly intubated, using a random number generator, in the trachea or in the esophagus. The presence or absence of the SLS was confirmed before each trial by the investigators.	Correct identification in 46 out of 48 trials. At the 9-month follow up the presence of absence of SLS was identified in 56/56 trials resulting in sensitivity and specificity of 100%.
Press et al. 2013 [24]	Didactic & practical	Application, knowledge & interpretation	2 h lectures & hands-on training followed by real patient training over 6 weeks	Baseline knowledge was ascertained via a pre-test before a 2-h lecture. In an instructor-participant ratio of 1:4 participants completed a FAST on different models. The second stage of training occurred over a 6-week period. APPs in groups of two attended a 4-h practical session with one of three emergency ultra-sonographers. APPs performed a minimum of four EFAST with supervised instruction on real patients. Six Web-based educational modules, 10–20 min in length progressively covered the techniques of EFAST scanning. Three weeks into the second phase of training, APPs practiced EFAST on trauma patients in flight. Finally, APPs attended a 1-h classroom lecture reviewing EFAST techniques and imaging. The pre-training test was re-administered and an OSCE was administered immediately after the post-training test.	Mean score for online module pretest was 43% (0 of 33 passed). Mean score post-test 78% (28 of 33 passed) 79% passed OSCE first attempt
Quick et al. 2016 [8]	Didactic & practical	Application & interpretation	Single series of lectures followed by real time US examinations.	Training consisted of didactic series of lectures, followed by real-time US examinations of the thorax on healthy human volunteers. Live animal models; (swine) were also utilised to visualise both normal and abnormal thoracic US findings.	Pre-hospital sensitivity of 68% and specificity 96% compared to sensitivity of 84% and specificity of 98% in emergency department of same patients. Aeromedical accuracy 91% vs surgeon 98% in the diagnosis of pneumothoraces.
Roline et al. 2013 [27]	Didactic (online) & practical	Application & interpretation	15 min online lecture + 60 min hands-on training	Care providers reviewed a standardized 15-min lecture online, which incorporated a review of thoracic ultrasound followed by a 60-min hands-on training session on healthy models during flight in the supine position. Participants evaluated the presence or absence of the sliding lung sign and recorded 6-s video clips of each side of the chest during that time.	58% of patients had thoracic US. Substantial agreement (kappa = 0.67) between helicopter operator and expert reviewer who looked at images later. Reviewer rated 54% of the images taken as ‘good’ quality.
Ulneur et al. 2011 [18]	Didactic & practical	Application & interpretation	4 h didactic training + 4 h hands-on training	Training was provided by a radiologist to conduct a FAST assessment. Results were recorded as positive/negative for free fluid in each case. Following training, 127 patients were evaluated by the paramedics. Patients then underwent abdominal US by radiology specialists who were blind to the study protocol but not to the clinical status of the patients. Computerized abdominal tomography (CAT) was ordered as desired by general surgeon consultants and evaluated by radiologists who were blind to the study. The gold standard for the presence of free fluid was the official radiologist reports of USG and CAT.	Paramedic performed FAST: sensitivity 84.62%, specificity 97.37%
West et al. 2014 [19]	Didactic & practical	Application & interpretation	4-h course involving both lecture and hands-on training	Training consisted of a 4-h course taught by a certified ultra-sonographer and board certified emergency physician. The course involved both lecture and hands on portions with access and training on both control and positives. After the training course a 2-week waiting period was allowed to lapse prior to simulation testing.	False-positive rate of 59% significantly higher than the false-negative rate of 41% (*p* < 0.01). Overall sensitivity of FAST scan in MCI was 67% and specificity of 56%. Average 121.8 s per exam.
Walcher et al. 2010	Didactic & practical	Application & interpretation	1-day course	Participants were introduced to the concepts of US and FAST in trauma. During three practical sessions, participants performed FAST under the supervision of experienced clinical instructors with a ratio of 1:2. Each participant performed up to 30 ultrasound procedures. Initially, participants performed the standardised procedure of FAST on both healthy volunteers and patient volunteers. Participants then learnt how to perform the ultrasound procedure under difficult circumstances. Finally, real-time scenarios of healthy or patient volunteers found in critical situations following an accident were presented. During the study period of 12 months, FAST investigations were performed on-scene and later evaluated for time and accuracy. The accuracy of the findings were verified using FAST and CT scanning in the emergency department as the gold standard.	Results from the 9 participants (C-group) compared with results from 2 other groups: P-group (10 trauma surgeons trained in FAST with <3 years experience) & I-group (9 ED flight physicians using US occasionally but not formally trained) After training C-group achieved 100% accuracy (in 39 procedures) No significant difference between C group vs P group or I group
Vitto et al. 2015 [25]	Didactic (including online component) + practical	Knowledge & application	6 h US training course over 4 month period	Participants completed a pre-test followed by a 90-min didactic lecture and 90-min hands on simulation session. Following this they were given 24/7 access to the POCUS. Four months following initial training the participants completed another 90-min didactic lecture and 90-min hands on simulation before completing a post-test questionnaire and survey.	Pretest and post test scores were 78% and 85% respectively.
6. *Consultation*
  Consultation was not included in this study due to time constraints.

## Results

The initial search yielded 2002 de-duplicated articles. The titles of the relevant articles were screened for inclusion, and 1,984 were excluded as per the study protocol, see Fig. 1. The final inclusion consisted of 18 articles. Characteristics of the included studies are provided in Table 2 and the educational details of each study is provided in Table 3.

### Participants professional backgrounds

The population of participants undergoing POCUS training varied. Most articles reported combined cohorts of emergency providers with varying years of experience, however most were ultrasound naïve. Eight articles (44%) described paramedic-only participants with small sample sizes of up to 20 [14–21]. One prospective cohort study described a larger paramedic population of 70 paramedics and 20 intermediate emergency medical technician (EMT) providers [22]. Five articles explored combined cohorts of flight paramedics and nurses [7, 8, 23–25]. One of these studies described 2 participants with prior, although limited, US exposure [7]. Only two articles reported on physicians and paramedics concurrently [6, 26]. One study reported on a ‘flight crew’ but did not differentiate the professional backgrounds of participants [27].

### Clinical assessments

There were six different clinical assessments for which paramedic POCUS curricula was explored, of which the FAST examination was the most common (Table 2).

### Clinical assessments and available curricula

The curricula varied from two-minutes to two-days, with some studies also reporting ongoing training of up to six weeks (Table 3). The methods used ranged from online home based study to face-to-face lectures or instructional sessions. Didactic and practical sessions were reported in all studies, with only one study reporting an additional 1-h online home-based study program prior to initial training [22]. One study completed another educational session including a 90-min lecture and 90-min hands-on four months following initial training [25].

To determine the impact of the education multiple methods including written tests, recognition and interpretation of images, and objective structured clinical examinations (OSCEs) were used (Table 3). A pre-test was conducted in three studies to determine baseline knowledge with improved post-test scores (43% vs 78%, 78% vs 85%, and 73% vs 95% respectively) following the educational program [22, 24, 25].

The POCUS was for the majority performed in simulation and on healthy volunteers, see Table 2. Heegard et al. was the only article to include a further 8-h of supervised US training on real patients in an emergency department [7]. Assessment for this study was conducted 6-weeks following initial training and involved a written test with an average score of 82% pre-training versus 71% when performed 12-months later. Regarding the performance of the paramedics, of the seven articles that reported on accuracy measures, sensitivity ranged from 67 to 97.5%, and specificity from 56 to 97% [7, 8, 17–19, 23, 25]. The lowest ranges related to a study in mass casualty incidents [19]. Results for a complete FAST examination was only reported by one author with a sensitivity of 84.6% and specificity of 97.3% [18]. One study specifically reported a sensitivity and specificity of 100% for pericardial effusion as well as a sensitivity of 60% and specificity of 93% for abdominal trauma cases [7].

The only study to report on the time taken to perform the assessment was the use of FAST in mass casualty incidents [19]. On average this took 121.8 s per examination [19].

Curricula for detecting pneumothoraces was described in four articles [8, 15, 23, 27]. Duration varied with the shortest session a 25-min combined instructional and practical program, and the longest a two-day program, see Table 3. All studies utilised didactic training followed by real-time practical ultrasound on either cadavers, healthy models, swine models, real patients or a combination of each. The sliding lung sign was described in three studies for identifying the presence or absence of pneumothorax [8, 24, 27], while one study also reported on comet tail artefacts, and seashore and stratosphere signs [15].

The sensitivity and specificity of POCUS for detecting pneumothoraces in the out-of-hospital setting following education was described by one study [8]. Quick et al. reported a sensitivity of 68% and a specificity of 84% compared to sensitivity of 84% and specificity of 98% in emergency department US of the same patients [8]. The accuracy of flight nurses and paramedics was found to be nearly as accurate (91% vs 98%) as the surgeon in the diagnosis of pneumothoraces [8]. At 9-months follow up Lyon et al. reported the highest sensitivity and specificity of 100% on a re-test for the identification of the sliding ling sign following a 25-min instructional video [23].

POCUS curricula for cardiac evaluation was explored in four articles [14, 16, 20, 21]. All studies included didactic and practical training, and duration varied from 2-h to 6-h. Booth et al. reported 88% of paramedics obtained successful views on healthy patients with improved theoretical knowledge from 54% pre-course to 89% post-course [14]. Only one study reported in-field use of cardiac evaluation where paramedics were able to obtain adequate scans 89% of the time [21]. Two cardiac arrests were logged and paramedics correctly identified both cases as cardiac standstill. Chin et al. explored combined curricula of the heart and lungs to evaluate for pneumothorax, pericardial effusion and cardiac activity [16]. An average test score of 9.1/10 on image recognition was reported, however 6 paramedics were unable to identify cardiac standstill.

POCUS curricula for long bone fracture recognition was reported by one study [17]. A fracture simulation model was composed of five different types of mechanically fractured bare turkey leg bone. Paramedic participants received a 2-min standard orientation and training session prior to evaluation. Across all fracture patterns the paramedics achieved an overall detection sensitivity of 97.5% and specificity of 95.0% [17].

Only one study reported on the POCUS curricula in the use of stroke assessment over a two-month period including theoretical training and real life training under neurological supervision [20]. This study remains ongoing and results are yet to be reported. Similarly, only one article explored POCUS curricula for peripheral intravenous access to determine if this decreases the use of central lines in the ED [28].

The use of POCUS to identify correct endotracheal tube placement was reported by one article [26]. In this study all participants intubated cadaver models without POCUS guidance followed by a 10-min lecture and hands-on training. Participants then intubated four cadaver models using POCUS guidance. Correct tube placement improved from 87.1% without POCUS guidance to 95.3% (*P* = 0.018) with POCUS guidance.

### Study location

Most of the studies were conducted in the United States (13 studies), while the remaining studies were undertaken in other countries including the United Kingdom (2 studies), Germany (2 studies) and Turkey (1 study), see Table 2.

## Discussion

Ultrasound is a relatively new and emerging assessment modality in out-of-hospital care, especially for paramedics. Technology has progressed to the point where small hand-held devices are readily available for out-of-hospital use. This scoping review examined 18 articles to describe the different curricula used for training paramedics in POCUS in the out-of-hospital setting.

The professional backgrounds of participants undergoing out-of-hospital POCUS training varies. Most studies reported on combined cohorts of physicians, nurses and paramedics all with varying years of experience. Knowledge of gross and functional anatomy can vary significantly, dependent on individual professional background (if any), and the duration and quality of initial education. Numerous physician-based prehospital services across the developed world have been using prehospital US for many years and are mostly helicopter emergency medical services (HEMS) [29, 30]. Physicians staffing EMS prehospital systems are usually sourced from the specialties of anaesthesia, intensive care and emergency medicine. Thus, these staff will already have undertaken significant education in gross and functional anatomy during undergraduate medical studies, and will likely have at least basic training in POCUS as part of their specialist qualification.

Conversely, paramedic regulation and registration as health professionals varies across the world, and there is no standardised qualification to be termed a paramedic in many jurisdictions [31]. The base qualifications for paramedics working in out-of-hospital systems in developed nations may range from short vocational courses, or undergraduate degrees through to postgraduate-level courses. Therefore, it is intuitive that experienced practitioners would gain and achieve competency in a skill dependent on anatomical knowledge more readily than less-experienced colleagues. In this study, paramedics with advanced training and/or extensive experience demonstrated a higher degree of accuracy in POCUS interpretation (where measured) [23]. However, the data available at this time does not show a trend towards a difference between the groups, and a correlation to clinical outcomes is not able to be demonstrated, and is beyond the scope of this study.

This scoping review suggests that the curricula used has varied considerably. Training duration ranged from a short 2-min orientation session to two days with most sessions completed over a one-day course. Of interest, the literature reports POCUS curricula being implemented for physicians as early as the first year of medical school [32]. In one study, Golgalniceanu et al. enrolled third and fifth year students in a 5-h FAST course of which 85% of students completed a full FAST scan at an adequate level of performance in under 6-min [33]. Should further studies demonstrate clear clinical utility of prehospital POCUS, then it may be reasonable to commence basic training in ultrasound during undergraduate paramedic training. Introduction of the skill early in the career of paramedics may strengthen anatomical knowledge, and allow for development of competence in POCUS over an extended period.

Blended learning that integrates face-to-face learning with computer-mediated online components has recently been reported as an effective approach for efficient learning and skill acquisition in POCUS [32, 34]. In this scoping review only two studies included an additional online component [22, 27]. Knapp et al. reported improved post-test scores from 73 to 95% with EMS providers scoring 98.9 to 99.1% on the OSCE stations [22]. This suggests online learning may assist with proficiency in learning the skills and knowledge required for performing POCUS, however is yet to be demonstrated as a superior learning tool. Simulation training for skill acquisition is also well documented in the literature [32, 35]. Interestingly, nine of the studies in this review were performed solely in the classroom on healthy volunteers or cadavers [6, 14–17, 19, 23, 25, 26]. The remaining studies were performed on ill or injured patients in clinical practice. Further research is required to demonstrate which method provides greater outcomes in regards to clinical education.

At the commencement of training for the various groups, strict protocol for the clinical application of POCUS was not always defined. After training was completed, actual POCUS utilisation varied from FAST, pneumothoraces, cardiac evaluation or a combination of these. The study by Chin et al. was robust in that it followed a strict protocol, although that protocol was limited to only cardiac and lung ultrasound [16]. Nevertheless, adhering to a structured, measured and comprehensive training program, and then instituting a systematic protocol for in-field application of POCUS allowed for ready analysis of outcomes. These inconsistencies may translate to difficulty in assessing clinical utility of out-of-hospital POCUS, but also how proficient paramedics were in interpreting images, and advancing their psychomotor skills.

Paramedic interpretation of ultrasound images in most studies was “confirmed” by US experts. However, in one study, there was no “closing of the loop” with regard to the learning process [18]. Correlation with expert interpretation would seem prudent, given the novel and relatively complex nature of POCUS in the out-of-hospital environment. This highlights the importance of studies examining paramedic education and proficiency in out-of-hospital POCUS which should include assessment of paramedic-acquired images by ultrasound experts, such as sonographers and/or experienced critical care physicians.

Despite various education approaches our review suggests that paramedics may be able to gain proficiency in POCUS reasonably promptly, regardless of base qualification, experience, duration or perceived quality of training. In the study by Quick et al., a small group of paramedics with (on average) greater than ten years’ experience, who underwent a comprehensive education and training program adhered to a strong study protocol and had a high level of patients exposure were able to demonstrate POCUS interpretation accuracy similar to that of trauma surgeons [8]. These data correlate with other studies where paramedics have initiated critical care procedures historically only performed by physicians (in and out-of-hospital) [36]. This suggests that a short duration of education and subsequent continuing clinical exposure (of which a minimum standard is difficult to determine) may be sufficient to enable paramedics to remain proficient in out-of-hospital POCUS. This concept requires further rigorous investigation through well-defined prospective clinical trials.

### Future directions

At this time, clinical utility of POCUS, especially in the hands of paramedic clinicians, has been difficult to demonstrate [37]. Further investigations may consider comparing basic out-of-hospital providers with critical care practitioners, who undergo a standardised POCUS education process. Such a study should examine not only experience of the clinician and the ability to interpret images, but also the level of exposure to the skill, and subsequent clinical application of information gained from POCUS exam.

A multi-center study, utilising a standardised education process with confirmation of image interpretation would be valuable to accurately measure paramedic proficiency. Additionally, such a study should assess variables including time taken to perform the scan, diagnosis and patient outcomes, with a view to evaluate clinical utility, and quantify the learning process. Other studies comparing solely classroom-based training versus live patient training, or a combination, would be useful in determining the most effective way to educate paramedics in the use of POCUS. In this scoping review, the studies did not compare a standard POCUS assessment. Future studies, such as a suggested standardised multi-center trial, should include standardised assessment so that accurate comparisons between groups can be made.

### Limitations

The authors acknowledge the limitations of the scoping review methodology. BM, NR and TSc are all operational paramedics utilising POCUS in their critical care practice, thus there is an acknowledged risk of bias in article selection and interpretation. This was mitigated by including authors that have limited or no experience in the application of out-of-hospital POCUS.

## Conclusions

POCUS curricula for paramedics in the out-of-hospital setting varies considerably. There appears to be no consistent approach, with some systems not quantifying or revising quality of learning. Paramedics with different levels of training and experience appear to be able to gain skills in POCUS, however correlation with clinical utility is difficult to measure. Furthermore, a lack of standardised training and a structured clinical application process makes it difficult to evaluate POCUS as a useful modality in the provision of out-of-hospital care. Further studies investigating education for paramedic POCUS and subsequent clinical application should include a standardised education process, expert assessment of paramedic competency, and formalised protocols for the application of out-of-hospital POCUS, so as to measure clinical utility.

## Abbreviations

eFAST: extended Focused Assessment Sonography for Trauma
EMS: Emergency Medical Service(s)
EMT: Emergency Medical Technician
FAST: Focused Assessment Sonography for Trauma
HEMS: Helicopter Emergency Medical Services
OSCEs: Objective Structured Clinical Examinations
POCUS: Point-of-care ultrasound
